# Pediatric Parotid Chronic Sclerosing Sialadenitis in an African-American Female: A Rare Case and Review of the Literature

**DOI:** 10.7759/cureus.8846

**Published:** 2020-06-26

**Authors:** Eytan Keidar, Jacob Shermetaro, Gary Kwartowitz

**Affiliations:** 1 Otolaryngology - Head and Neck Surgery, McLaren Oakland Hospital, Pontiac, USA; 2 Medical Education, Michigan State University, East Lansing, USA

**Keywords:** küttner tumor, chronic sclerosing sialadenitis, parotid, malignancy, igg4 related disease, igg4 related sialadenitis

## Abstract

Chronic sclerosing sialadenitis (CSS) or Küttner tumor is an under-recognized, benign fibroinflammatory disease most commonly seen in the submandibular gland of older adult males. Sialolithiasis or bacterial infection was first suspected as an etiology, but CSS is now considered an immunoglobulin G4-related disease (IgG4-RD). IgG4-RD can affect almost every organ in the body, characterized by organ fibrosis with IgG4-positive plasmacytes. Numerous autoimmune-related diseases have been unified under IgG4-RD, including Mikulicz disease (MD), autoimmune pancreatitis, Reidel’s thyroiditis, and others. In any organ, IgG4-RD can present similar to malignancy. Due to the ability to mimic malignancy, it is crucial to be aware of this under-recognized clinical entity. CSS is currently of broad and high clinical interest due to increased understanding, multiorgan involvement, and more clearly defined criteria. To increase awareness of this disease, we describe a rare presentation of CSS with a literature review.

## Introduction

Chronic sclerosing sialadenitis (CSS) or Küttner tumor is an uncommonly recognized, benign fibroinflammatory disease of the major salivary glands. It primarily occurs in the submandibular gland of middle-aged and elderly adults, typically affecting males more than females [1]. Immunoglobulin G4-related disease (IgG4-RD) was first described as sclerosing (or autoimmune) pancreatitis. IgG4-RD can affect almost every organ in the body and is characterized by organ fibrosis with infiltration of IgG4 secreting plasmacytes [2]. The nomenclature of CSS has been proposed to be “IgG4-associated sialadenitis” or, more recently, “IgG4-associated chronic sialadenitis” [3-4]. Numerous autoimmune-related diseases have been unified under the umbrella of IgG4-RD, including Mikulicz disease (MD), autoimmune pancreatitis, retroperitoneal fibrosis (Ormond’s disease), Reidel’s thyroiditis, and others [5].

Although the majority of pediatric salivary gland tumors are benign, when a parotid mass is discovered in a pediatric patient, it can be a more ominous sign. This is due to the higher rate of salivary gland malignancy, roughly 15%-32% when compared to adults [6].

CSS is currently of broad and high clinical interest due to increased understanding, multiorgan involvement, and more clearly defined criteria. Karim et al. presented the first dedicated pediatric literature review of IgG-RD in any organ in order to highlight the fact that IgG4-RD is not limited to adults [7]. In an attempt to further increase awareness of this underrecognized disease, we present, to the best of the author's knowledge, the youngest female presenting with isolated CSS of the parotid gland. A narrative review of the literature is presented as well.

## Case presentation

**Figure 1:**
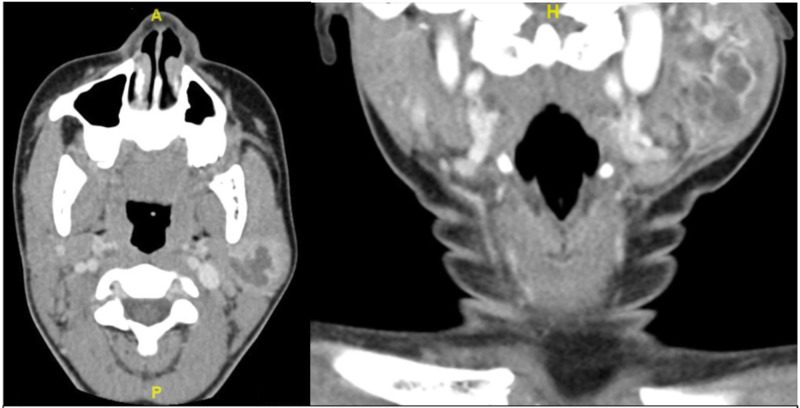
Computed tomography (CT) images of chronic sclerosing sialadenitis (CSS) of the left parotid gland in a pediatric patient Axial (left) and Coronal (right) computed tomography images of revealing loculated, cystic, left parotid mass ~6 x 4 x 3cm.

A 15-year-old African-American female presented to the community otolaryngology clinic for the evaluation of a left neck mass, progressively enlarging over the past 10 months. The patient stated that the mass was firm and largely non-tender with periods of sensitivity, mildly relieved with nonsteroidal anti-inflammatory drugs (NSAIDs). She denied fevers, chills, weight changes, or any other masses in the head and neck. The mass failed to respond to multiple courses of antibiotics prescribed by her primary care physician. Past medical history was reported as unremarkable. On physical exam, there was a 4 x 3 cm mass overlying the left mandible angle and ramus, which was mobile, soft, and swollen appearing. Contrasted computed tomography (CT) was obtained, which revealed a large, loculated, cystic, parotid mass ~6 x 4 x 3cm (Figure 1). Fine needle aspiration (FNA) was performed, revealing scant cellularity, negative for malignant cells as well as histocytes, and normal salivary glands.

**Figure 2:**
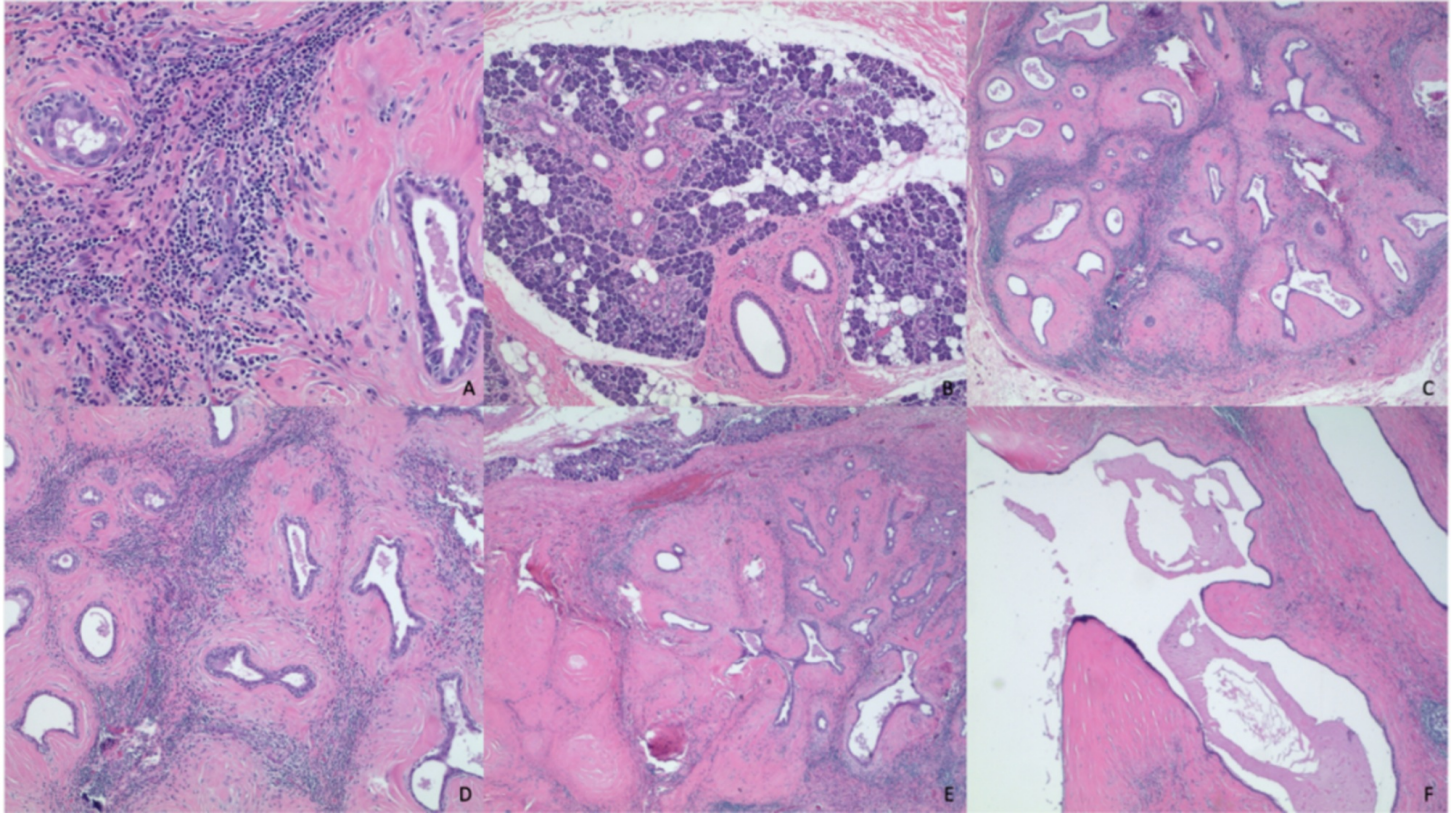
Sections of the left parotid gland and lymph node stained with hematoxylin and eosin Sections of the left parotid gland and lymph node with hematoxylin and eosin-stained tissue showing marked chronic sclerosing sialadenitis. A - Section of the tumor with moderate chronic inflammatory infiltrates composed predominantly of lymphocytes with some plasma cells; B - Normal parotid salivary gland; C - Salivary gland tissue retaining the lobular architecture with marked fibrosis, acinar atrophy, and duct dilation; D - Section of the tumor with chronic inflammatory infiltrates; E - Section of the tumor with some ducts showing squamous metaplasia; F - Section showing a dilated duct filled with mucinous debris

A chronic inflammatory process, such as sialadenitis, was suspected and surgical removal recommended. During parotidectomy, the tumor involved the deep lobe and was successfully removed, without complications. Pathology revealed a 6.5 x 4 x 3 cm grossly multiloculated cystic mass containing mucin-like material and a benign lymph node showing dermatopathologic lymphadenitis. On microscopic exam, the tumor showed a retained lobular architecture with marked fibrosis, acinar atrophy, and duct dilation along with moderate chronic inflammatory infiltrates of lymphocytes and some plasma cells. Some ducts showed squamous metaplasia. These findings are consistent with those of chronic sclerosing sialadenitis and are presented in Figure 2.

## Discussion

IgG4-RD can lead to significant morbidity and/or mortality in untreated patients [8]. IgG4-RD commonly involves the salivary glands and can be involved in 27%-53% of IgG4-RD’s, typically with a bilateral presentation [9]. IgG4-RD can involve the orbits, lymph nodes, thyroid, sinonasal cavities, and pituitary gland. Data suggest the pediatric population presents with an orbital disease 44% of the time and salivary gland involvement less than 28% of the time [7].

Presentation

Although there have been reports of unilateral parotid gland involvement of CSS, its presentation is rare, especially in pediatric African-American patients [10-12]. Due to the relatively novel status of CSS within the umbrella of IgG4-RD, there is no meaningful epidemiologic data. In the pediatric literature search by Karim et al., a 10-year-old female was included [7]. However, the disease involved the submandibular, parotid, and lacrimal glands. Culver et al. proposed an algorithm when evaluating an enlarged salivary gland (unilateral or bilateral), which included obtaining serum IgG4 and performing ultrasound-guided FNA when clinically appropriate [13].

Differential diagnosis

The differential diagnosis of CSS typically includes sialolithiasis-induced sialadenitis, autoimmune processes, such as granulomatosis with polyangiitis (GPA or formerly Wegner granulomatosis), Sjögren's disease, sarcoidosis, and neoplastic processes such as lymphoma and carcinoma [14]. The histopathologic features of CSS differ from sialolithiasis-associated sialadenitis by the presence of cellular fibroinflammatory areas composed of fibroblasts, lymphocytes, and plasma cells that can be used to distinguish CSS [1]. Autoimmune or systemic diseases typically have a bilateral presentation as demonstrated in Sjögren's syndrome but can present with salivary gland and multiorgan involvement [2]. Due to these similar possible clinical presentations, without a high index of suspicion, diagnosis is difficult and additional testing is typically required.

Imaging

Although the diagnosis of CSS is difficult with imaging alone, there have been reports of diagnosis solely through sonography [10]. CSS can be focal or nodular, mimicking neoplasia, but it is difficult to diagnose with imaging alone. Sonography is typically used in conjunction with biopsy [13]. CT may be done to visualize the full mass and identify subclinical disease. Characteristic findings on imaging include diffuse and focal organ infiltration with encasement by inflammatory and fibrotic tissue, however, these findings are non-specific [14].

Cytology, histology, and immunohistochemistry

In CSS, FNA findings may be non-specific but can suggest CSS in the appropriate clinical setting [15]. Due to the low specificity of FNA in CSS, clinicians use it to excluded malignancy [13]. FNA of a neck mass showing squamous metaplasia and salivary tissue lacking acini should push the diagnostician to further evaluate and rule out a malignant process [16].

CSS was originally differentiated into four stages based on histologic features, with stage 4 being the loss of lobular architecture, which is what this histology indicates [17]. Later on, the diagnostic criteria of IgG4-RD of any involved organ (Table 1) were proposed by Umehara et al., including clinical findings as well as serological and immunohistochemistry (IHC) testing of IgG4 [18]. However, due to the characteristic histology of CSS, the 2011 Boston consensus statement identified three major histopathologic features associated with IgG4-RD. These include (1) dense lymphoplasmacytic infiltrate; (2) fibrosis, arranged at least focally in a storiform pattern; and (3) obliterative phlebitis. Other features include phlebitis without obliteration of the lumen and increased numbers of eosinophils. More importantly, it is asserted that a confident pathological diagnosis of IgG4-RD can be made with the histologic diagnosis alone such as with this case presented [4]. In a smaller community hospital setting, IgG4 staining may not be available.

**Table 1:** Diagnostic criteria of IgG4-RD proposed by Umehara et al. Source: [18] IgG4-RD: immunoglobulin G4-related disease; IgG: immunoglobulin G

Diagnostic criteria of IgG4-RD									
1. Clinical examination showing characteristic diffuse/localized swelling or masses in single or multiple organs									
2. Hematological examination shows elevated serum IgG4 concentrations (135 mg/dl)									
3. Histopathologic examination shows:									a) Marked lymphocyte and plasmacyte infiltration and fibrosis.
									b) Infiltration of IgG4+ plasma cells: ratio of IgG4+/IgG+ cells > 40% and >10 IgG4+ plasma cells/HPF
Definite: 1, 2, and 3									
Probable: 1 and 3									
Possible: 1 and 2									

Therapeutic options

Conservative treatment has been proposed versus surgical. However, corticosteroids are the first line of treatment for IgG4-RD with a good prognosis; some report up to 61% remission at one year, but relapse is not uncommon, especially when steroids are discontinued [5]. Dosing with prednisolone 0.6-1.0 mg/kg daily that is tapered down after two to four weeks by 5 mg every one to two weeks has been proposed [19]. Rituximab, an anti-CD20 monoclonal B-cell inhibitor, can be used in steroid-intolerant patients and has shown to be effective as well [20]. In patients who acquire relapse or refractory disease, third-line immunosuppressive agents include thiopurines and mycophenolate, although they have not been shown to be as effective [7].

## Conclusions

CSS or Küttner tumor is currently considered to be a manifestation of IgG4-RD, which can present in several different organs solitarily or as part of multi-organ systemic disease. CSS most commonly presents bilaterally in the submandibular glands of middle-aged males. It is imperative to differentiate this disease from ominous malignancy. A multidisciplinary team is necessary and the patient should be referred to a rheumatologist and possibly a hematologist. A strong pathologic diagnosis can be made with histology alone. Additionally, as awareness of IgG4-RD continues to increase, its recognition and diagnosis is likely to follow.
